# Development of *Prosopis juliflora* carbon-reinforced PET bottle waste-based epoxy-blended bio-phenolic benzoxazine composites for advanced applications

**DOI:** 10.1039/c9ra08741a

**Published:** 2020-02-04

**Authors:** V. Selvaraj, T. R. Raghavarshini, M. Alagar

**Affiliations:** Nanotech Research Lab, Department of Chemistry, University College of Engineering Villupuram (A Constituent College of Anna University, Chennai) Kakuppam Villupuram Tamil Nadu India rajselva_77@yahoo.co.in vaithilingamselvaraj@gmail.com +91-4146-224500 +91 9003509320; Polymer Engineering Laboratory, PSG Institute of Technology and Applied Research Neelambur Coimbatore-641 062 India

## Abstract

An attempt has been made in the present work to develop hybrid blended composites using epoxy resin (PETEP) derived from waste polyethylene terephthalate (PET) bottles and bio-phenolic (cardanol)-based benzoxazine (CBz) reinforced with functionalized bio-carbon (f-PJC) obtained from *Prosopis juliflora* (PJ) for high performance applications. The molecular structure, thermal properties, thermo-mechanical behaviour, morphology, surface properties, and corrosion resistance of the composites were studied by different analytical methods, and the obtained results are reported. Dynamic mechanical properties such as the storage modulus (2.591 GPa), loss modulus (1.299 GPa) and cross-linking density (5.1 × 10^7^ J mol^−1^ K^−1^) were improved in the case of the 5 wt% f-PJC/PETEP–CBz composite compared to those of the PETEP–CBz blended matrix and the f-PJC/PETEP–CBz composites with other weight percentages. Among the studied bio-carbon-reinforced hybrid composites with different weight percentages, the 5 wt% f-PJC/PETEP–CBz composite shows a higher value of char yield (38.37%), with an enhanced glass transition temperature of 285 °C and an improved water contact angle of 111.3°. Results obtained from corrosion studies infer that these hybrid composites exhibit improved corrosion resistance behaviour and effectively protect the surface of mild steel specimens from corrosion. It is concluded that the present work can be considered as an effective method for utilizing waste products and sustainable bio-materials for the development of high performance value-added hybrid composites for thermal and corrosion protection applications.

## Abstract 2

Schematic representation of development of functionalised *Prosopis juliflora* carbon (f-PJC) reinforced PET-epoxy resin (PETEP) blended bio-phenolic (cardanol) based benzoxazine (CBz) hybrid composites for high performance applications.

## Graphical abstract

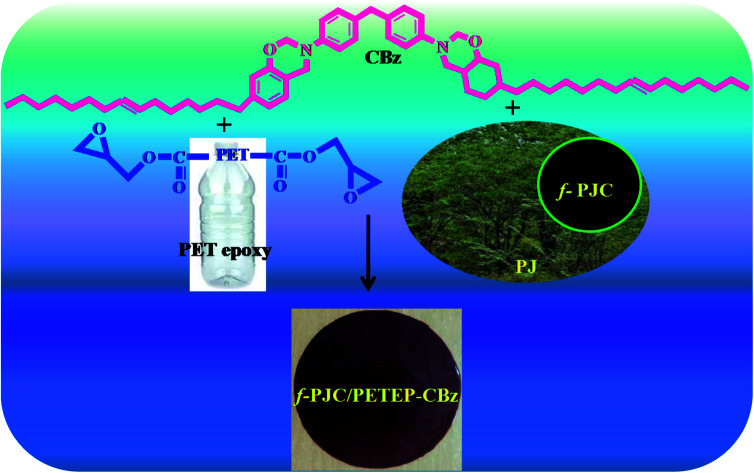

## 1. Introduction

Polyethylene terephthalate (PET) is considered to be one of the most important engineering polymers; it is extensively used in a variety of applications, including packaging in the form of films and bottles. PET possesses excellent mechanical strength, chemical resistance, clarity, transparency, processability, and colourability as well as reasonable thermal stability.^1^ PET recycling is one of the most successful and widespread examples of polymer waste utilisation. In addition, PET materials play an important role in the beverage and food industries.^2^ PET is also used in films,^3^ fibres,^4^ coatings,^5^*etc.* Hence, researchers worldwide have devoted focus on converting PET waste into value-added raw materials,^6^ non-fibrous materials,^7^ essential intermediates^8^ and other useful products. The utilization and conversion of PET waste into useful products will also be helpful in minimizing land, water and air pollution.^9^ Benzoxazine materials are known for their high temperature applications; they can be easily synthesized through ring-opening of cyclic benzoxazine monomers by a heat treatment process in the absence of any catalysts.^10^ Polybenzoxazines find a wide range of applications, *viz.*, composite materials,^11^ construction,^12^ aerospace,^13^ printed circuit boards,^14^ brake pad adhesives,^15^ packaging in the microelectronics industry,^16^ co-curing agents,^17^ and fire retardants.^18^

Further, the synthesized phenolic resins are resistant to solvents, moisture, heat and electrical conductance; they also have a variety of properties with a wide range of applications. However, synthetic phenolic resins have some drawbacks, such as by-product formation during condensation,^19^ high cost and environmental concerns.^20,21^ Mostly, phenolic benzoxazines are obtained from petroleum-based resources; they are more brittle than biobased materials with long aliphatic chains, whose substituents provide more toughness along with balanced brittleness. Therefore, researchers are shifting focus towards naturally occurring phenolic products, such as cardanol and eugenol, to avoid environmental pollution, to decrease brittleness and to improve toughness.^22^ Among the natural phenolic resins, cardanol is an important raw material of natural origin that is obtained by the vacuum distillation of cashew nut shell liquid. Cardanol and its derivatives play vital roles in industrial utilization, such as diesel engine fuels, additives for lubricants, pour point depressants, antioxidants, flame retardants, stabilizers, resins, hydro repellents, inks, intermediates and fine chemicals, including pharmaceutical applications.^23,24^

Similarly, activated carbons obtained from bituminous coal, lignite and peat are expensive. Meanwhile, agricultural wastes and residues are rich in lignocellulose and carbonaceous content; they are abundantly available and can be used for the production of activated carbon that is economically low in cost.^25^ Activated carbons are prepared by two important methods, namely thermal (pyrolysis) and chemical (acid or base) activation. In chemical activation, the acidic activated carbon can be prepared using phosphoric acid, zinc chloride, sulfuric acid, nitric acid, or a mixture of acids, while basic activation can be achieved with sodium hydroxide, potassium hydroxide or sodium carbonate. The activated carbons are expected to have large surface areas, lower surface energies, simple usage and high porosity with several functional groups.^26^

The chemical modification of activated carbon is an adaptable method to enhance their selectivity toward certain analyses. Activated carbon can act as a reactive intermediate to attach moieties, including amine and alcohol groups.^27^ Based on the increasing interest and environmental concerns, the present work proposes the synthesis and characterization of cardanol benzoxazine blended with PET bottle waste-based epoxy resins for the production of partial bio-composites; we are pursuing new strategies to reuse PET bottles to minimise the environmental impact of PET bottle waste.

*Prosopis juliflora* is a wild thorny plant that grows in wasteland; it naturally occurs in drought areas. It is well known that *Prosopis juliflora* wood-based carbon is dense, porous, and has a high surface area and good carbon content; it can be used in various applications, including thermal energy production. Hence, we chose *Prosopis juliflora* carbon and chemically modified it to achieve chemical compatibility with the polymer matrix. In this regard, *Prosopis juliflora* carbon was oxidised to create functional groups and then coupled with amino tetra ethoxy silane to obtain amine terminal groups through covalent bond formation, which have high reactivity and can be directly incorporated into the polymer matrix.^28^

With these views in mind, in the present study, we attempted to develop bio-phenol-based benzoxazine with an epoxy resin (PETEP) derived from polyethylene terephthalate (PET) waste bottles and epichlorohydrin in basic medium. Further, various weight percentages of bio-carbon derived from *Prosopis juliflora* (f-PJC) functionalized with triethylene tetramine (TETA) were reinforced with blends of the hybrid PET epoxy-cardanol benzoxazine matrix to obtain f-PJC/PETEP–CBz composites. The PETEP–CBz matrix and f-PJC/PETEP–CBz composites were characterized by different analytical techniques to assess their suitability for high performance applications, and the obtained results are discussed and reported.

## 2. Materials and methods

### 2.1 Materials

Cardanol (CAS 37330-39-5, 99%) was procured from Satya Cashew Chemicals Pvt. Ltd. Chennai, India. Epichlorohydrin (CAS 106-89-8, 99%), 4,4′-diaminodiphenylmethane (CAS 101-77-9, 99%), sodium hydroxide (CAS 1310-73-2, 99%) and triethylene tetramine (TETA) (CAS 121-44-8, 99.5%) were obtained from Sisco Research Laboratories, Pvt. Ltd. (SRL), India. Paraformaldehyde (CAS 30525-89-4, 100%) was acquired from Merck Limited, India. All other chemicals were of analytical grade and were used without further purification.

### 2.2 Synthesis of PET-epoxy from PET bottle waste

**Scheme 1:**
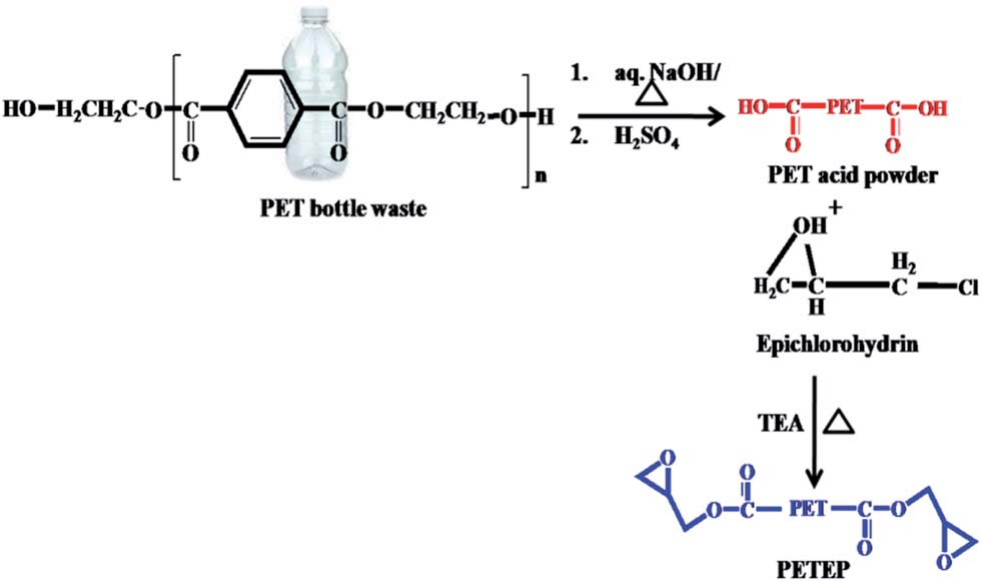
Synthesis of PET-epoxy from PET bottle waste through hydrolysis followed by an epoxidation process.

Initially, PET waste bottles were cut into small pieces. 10 g of sodium hydroxide dissolved in 45 mL water was added to 6 g of sample PET bottle waste. The reaction container was heated at 130 °C for 48 h. The resulting reaction product was poured into a beaker, followed by acidification with 20% hydrochloric acid; it was then filtered and dried in an oven at 60 °C for 24 h. The pre-treated PET sample was obtained as a white powder with a yield of 90% (Scheme 1).

9.6 g of pre-treated PET sample powder was mixed with 73 mL of epichlorohydrin and heated at 70 °C for an hour.^29^ Then, 0.6 mL of triethylamine was injected slowly, and the temperature was increased to 100 °C to 110 °C under reflux conditions; the same temperature was maintained for six days under stirring conditions. The product obtained was distilled under reduced pressure to remove excess epichlorohydrin. The viscous liquid was dissolved using dichloromethane and dried over sodium sulphate. The reddish brown PET-epoxy resin was obtained (Scheme 1) after distilling the solvent under vacuum.

### 2.3 Preparation of functionalized *Prosopis juliflora* carbon (f-PJC)

**Scheme 2:**
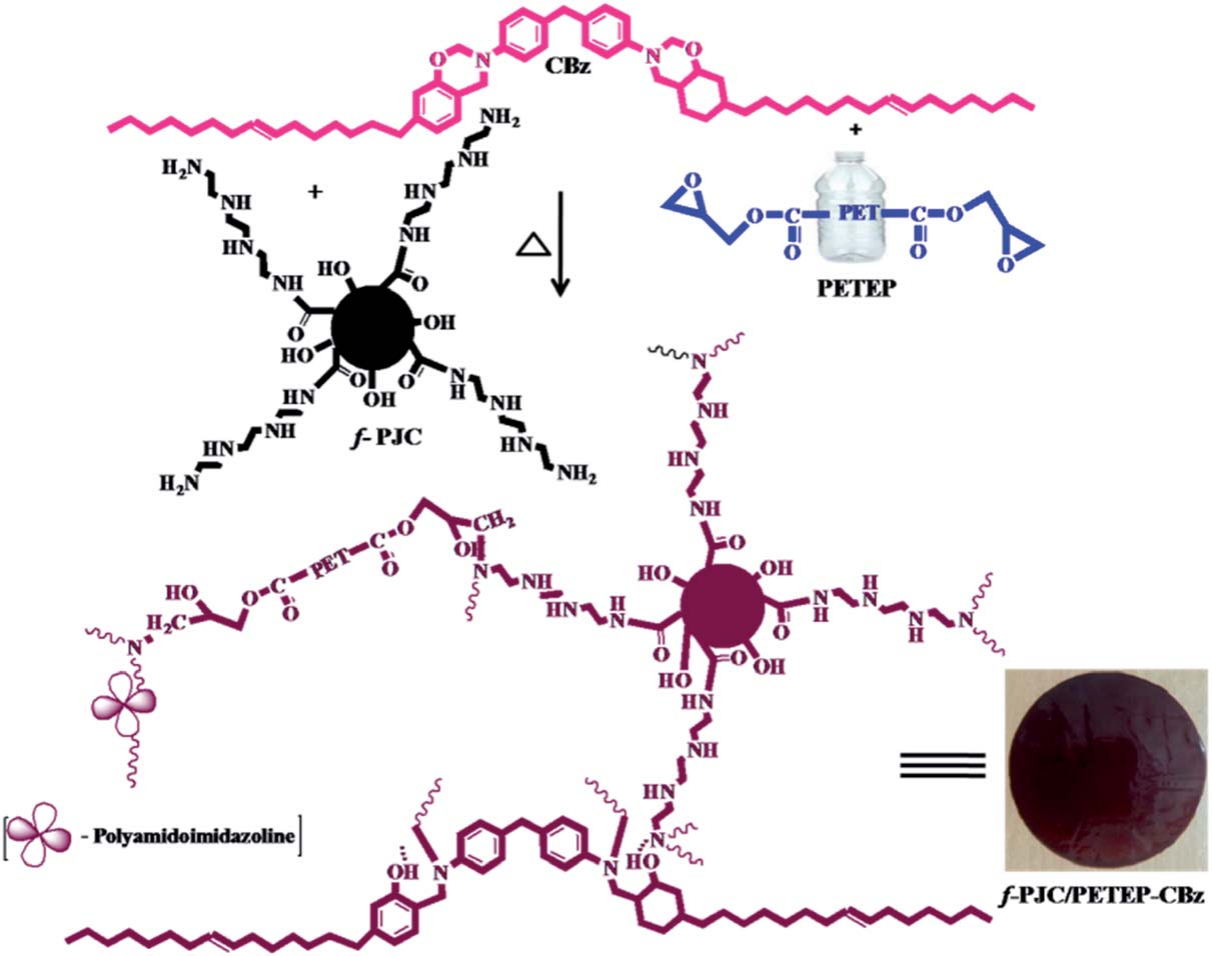
Preparation of the PETEP–CBz blended matrix and f-PJC-reinforced PETEP–CBz composites.

Initially, 15 g of sunlight-dried and chopped *Prosopis juliflora* stem was carbonized in a muffle furnace in a ceramic crucible at 400 °C for 5 h; it was then soaked in 1 : 3 ratio of HNO_3_ : H_2_SO_4_ under reflux conditions for 24 h.^30^ The resulting carbon was washed with water, and the washing process was continued until the filtrate became neutral; then, the resulting solid product was dried at 100 °C in a hot air oven for 48 h. 10 g of obtained *Prosopis juliflora* carbon was crushed into powder, and 1 wt% of sodium bicarbonate was added; the mixture was stirred for 24 h. Finally, the resulting carbonaceous material was filtered and washed to remove the salt, then dried at 100 °C for 24 h in a hot air oven to obtain carbonate-treated *Prosopis juliflora* carbon. 7 g of carbonate-treated *Prosopis juliflora* carbon was mixed with 30 mL of methanol and refluxed for half an hour. To this reaction mixture, 6 mL of triethylenetetramine (TETA) was added, and the mixture was refluxed under stirring for another 8 h. The solid material obtained was filtered, washed with methanol and dried at 100 °C for 24 h to afford amine-functionalised *Prosopis juliflora* carbon (f-PJC) (Scheme 2).

### 2.4 Synthesis of cardanol-based benzoxazine (CBz) monomer

14 g 4,4′-diphenyldiaminomethane (DDM) and 4 g of paraformaldehyde were taken in a 100 mL round bottomed flask and stirred at a temperature of 85 °C for an hour.^31^ 20 g of cardanol was added to the above reaction mixture, and the temperature was raised to 120 °C. The same temperature was maintained for 24 h in order to complete the reaction. Then, the reaction mixture was poured into 2.0 N sodium hydroxide, and the compound was extracted using chloroform. The organic layer was separated using a separating funnel, dried over anhydrous sodium sulphate and filtered. The chloroform solvent was removed under reduced pressure to afford cardanol-benzoxazine as a red-orange gel (Scheme 2).

### 2.5 Preparation of *Prosopis juliflora* carbon (f-PJC)-reinforced PET epoxy-cardanol-based benzoxazine composites (f-PJC/PETEP–CBz)

The *Prosopis juliflora* carbon (f-PJC)-reinforced PET epoxy-cardanol-based benzoxazine composites (f-PJC/PETEP–CBz) were prepared in accordance with Scheme 2. Typically, 2.0 g of cardanol-based benzoxazine (CBz) was mixed with 2.0 g of PET epoxy resin and 0.5 g of polyamidoimidazoline (Aradur-140) in 20 mL of chloroform, and then stirred to obtain a homogeneous solution. Simultaneously, varying weight percentages (1, 3 and 5 wt%) of *Prosopis juliflora* carbon (f-PJC) were added to the above reaction blend; then, the mixture was stirred to obtain a homogeneously dispersed product, which was then poured on a silane-coated glass plate and cured at 40 °C, 60 °C, 80 °C, 100 °C, 120 °C, 160 °C, 180 °C and 200 °C for one hour at each temperature to afford the flexible polymeric blended composite films (f-PJC/PETEP–CBz). Further, a PETEP–CBz blend matrix was prepared under similar experimental conditions.

### 2.6 Characterization

Fourier transform infrared spectra were recorded using a PerkinElmer spectrometer, and the vibration absorptions were recorded in the wave number range between 400 cm^−1^ and 4000 cm^−1^ for the PETEP–CBz matrix and its composites. The KBr pellets were fabricated using a pinch of PET powder, PJC and f-PJC compounds, while the PETEP–CBz matrix and varying weight percentages (1, 3 and 5 wt%) of f-PJC/PETEP–CBz composites were analysed in the form of films without KBr pellets. ^1^H NMR and ^13^C NMR spectra were acquired using a Bruker EXT40617 spectrometer at 25 °C using CDCl_3_ as the solvent. The morphological images were obtained using a Hitachi scanning electron microscope, Model S-3400N. Thermo gravimetric analysis (TGA) results were obtained using a NETZSCH TG 209F3 instrument. Differential scanning calorimetry (DSC) analysis was carried out using a NETZSCH DSC 214 Polyma. The hydrophobic natures of the composites were determined using an OCA EC15 contact angle instrument from Data Physics, GmbH, Germany. A dynamic mechanical analyzer (DMA, model DMS 6100) was used to determine the mechanical properties of the PETEP–CBz matrix and f-PJC/PETEP–CBz composites. The electrochemical Tafel plots were obtained using a Biologic VSP2 multichannel (France) workstation analyzer in order to predict the anticorrosion properties.

## 3. Results and discussion

### 3.1 FTIR studies of PET bottle waste powder and PET waste bottle-based epoxy resin

**Fig. 1:**
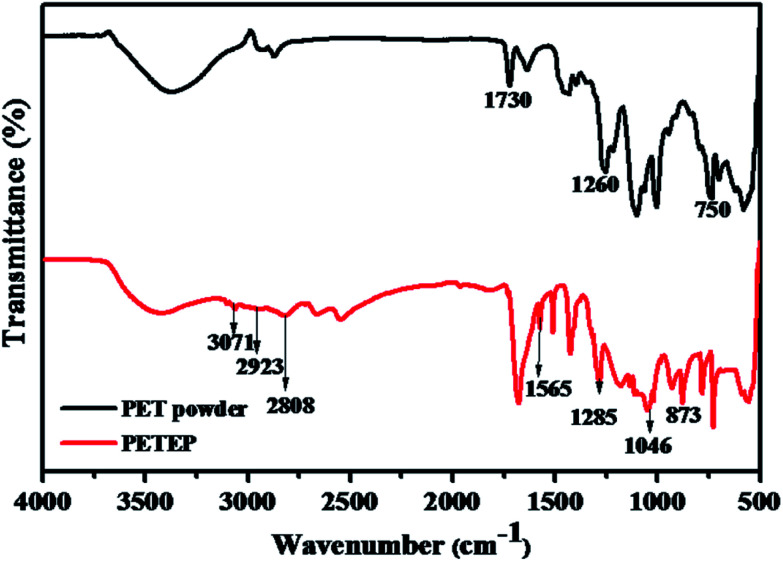
FTIR spectra of PET powder and the PET-epoxy (PETEP) resin.

FT-IR spectra of PET powder and the PET-epoxy resin are shown in Fig. 1. PET powder shows an absorption band between 3565 cm^−1^ and 3294 cm^−1^, which is ascribed to the absorption of –OH stretching vibration. From the FTIR spectrum of PET powder, the absorption peak corresponding to –C=O appeared at 1730 cm^−1^, along with the peak corresponding to the –OH stretching vibration; this confirms the presence of carboxylic acid functional groups in the PET powder. In addition, an absorption band was observed at 1260 cm^−1^, which is assigned to ester groups. The absorption bands appearing at 2939 and 2873 cm^−1^ are due to asymmetric and symmetric –CH_2_ stretching, respectively, along with the peak observed at 1260 cm^−1^ for ester groups; these confirm the formation of PET epoxy resin. Further, the characteristic absorption bands observed at 3071 cm^−1^ and 1565 cm^−1^ are due to the C–H and C=C stretching vibrations of the benzene ring.^32^ The appearance of the above absorption bands confirms the formation of PET functionalized with acid groups through the depolymerisation of samples of polyethylene terephthalate (PET) waste bottles.^33^ Along with the other peaks, the FT-IR spectrum of PET epoxy shows distinguishable stretching vibrations of C–O present in ester groups and oxirane ring –O–C and C–O–C bonds in the epoxy ring at 1285 cm^−1^, 1046 cm^−1^ and 873 cm^−1^, respectively; thus, the FTIR results infer the formation of PET epoxy resin.

### 3.2 FT-IR spectra of polybenzoxazine and its composites

**Fig. 2:**
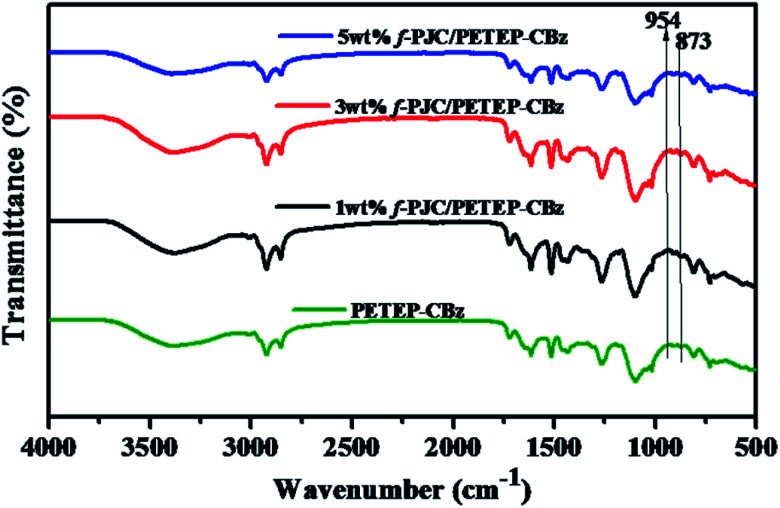
FT-IR spectra of the PETEP–CBz blended matrix and f-PJC/PETEP–CBz composites.

The changes in the molecular structures of the cured PET epoxy-cardanol-based benzoxazine-blended (PETEP–CBz) matrix and functionalized *Prosopis juliflora* carbon (f-PJC)-reinforced PET epoxy-cardanol-based benzoxazine (f-PJC/PETEP–CBz) composites were analysed using FTIR spectroscopy (Fig. 2). The characteristic absorption peaks that correspond to benzoxazine (C–O–C) ring and oxirane ring stretching mode vibrations at 954 and 873 cm^−1^, respectively, disappeared for the cured PETEP–CBz and f-PJC/PETEP–CBz composites.^34^ The absorption peak appearing at 1260 cm^−1^ is due to C–O stretching vibrations, and the broad absorption band observed at 3383 cm^−1^ was assigned to the –OH stretching vibrations produced during the curing process.^35^ In addition, the absorption peak appearing at 1104 cm^−1^ corresponds to symmetric C–O–C stretching, which is due to the ether linkages present in the PETEP–CBz matrix and f-PJC/PETEP–CBz composites.^36^ It was ascertained that the FT-IR studies confirm the formation of the PETEP–CBz matrix and varying weight percentages of f-PJC-reinforced PETEP–CBz composites.

### 3.3 Morphological studies

**Fig. 3:**
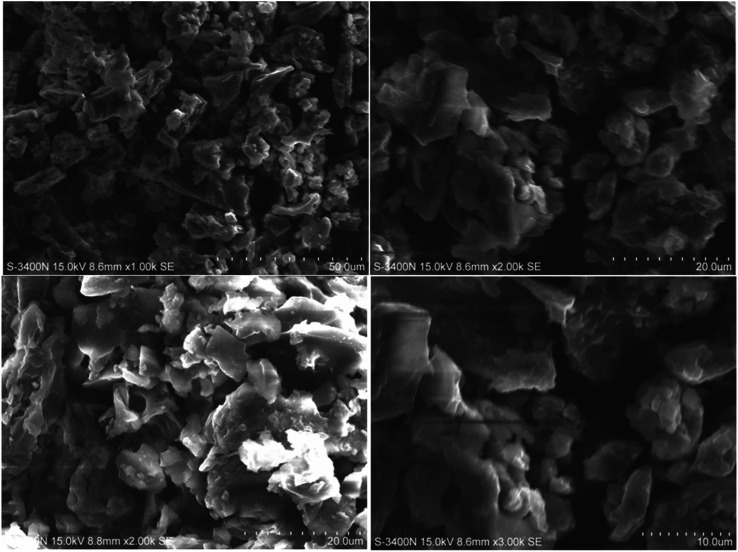
SEM images of amine functionalized *Prosopis juliflora* carbon (f-PJC) at various magnifications.

**Fig. 4:**
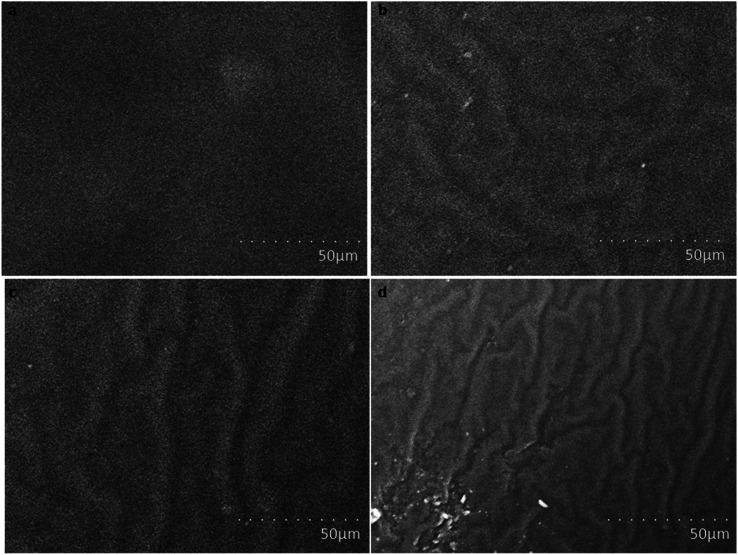
SEM images of (a) the PETEP–CBz matrix and the (b) 1 wt% f-PJC/PETEP–CBz, (c) 3 wt% f-PJC/PETEP–CBz and (d) 5 wt% f-PJC/PETEP–CBz composites.

Fig. 3 shows scanning electron microscopy images of amine-functionalized *Prosopis juliflora* carbon (f-PJC) at different magnifications. The SEM images of f-PJC at different magnifications indicate a sheet-like morphology randomly combined with folding and wrinkled surfaces along with distinct edges.^37–39^Fig. 4 shows SEM images of the PETEP–CBz matrix and its composites (f-PJC/PETEP–CBz). The PETEP–CBz matrix has a smooth surface (Fig. 4a), while reinforcing f-PJC in the PETEP–CBz matrix contributes to the development of a rough surface morphology. Further, the increased rough surface was accompanied with more ductile sunken areas, which influences the enhanced mechanical properties of the composites due to better interlocking between the molecules. In addition, it was observed that the river^40^-like surfaces become more concentrated with increasing f-PJC content, which may be due to the formation of improved cross-linking networks. Thus, more ductile sunken areas were noted for the 5 wt% f-PJC/PETEP–CBz composite compared to the prepared f-PJC/PETEP–CBz composites with other weight percentages. It is also inferred that the 5 wt% f-PJC/PETEP–CBz composite possesses a rougher surface morphology, more dimples and more ductile sunken areas, which are expected to contribute to high ductility and good mechanical properties.^41^

### 3.4 Thermal behaviour of PETEP–CBz matrix and its composites

**Fig. 5:**
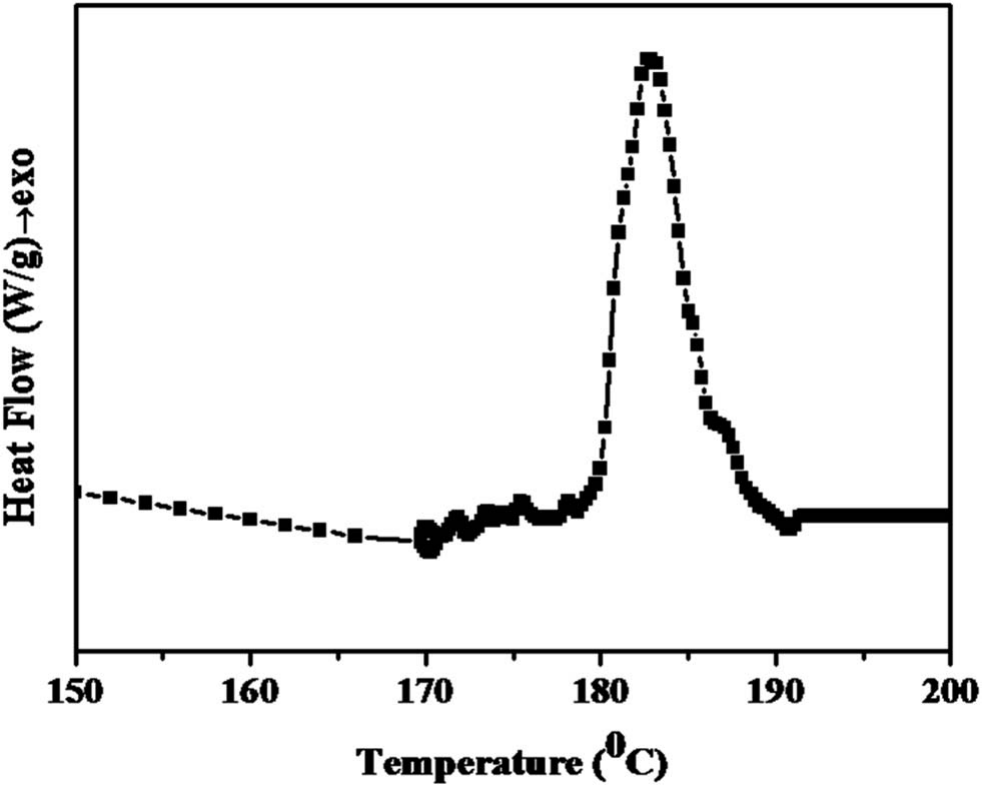
DSC curve for the PETEP–CBz pre-polymer mixture with the calculated quantity of polyamidoimidazoline as the curing agent.

Initially, DSC analysis was carried out for the PETEP–CBz pre-polymer mixture with the calculated quantity of polyamidoimidazoline to study the curing behaviour of the present system, and the DSC thermogram obtained for the synthesized PETEP-biobased cardanol benzoxazine hybrid pre-polymer is presented in Fig. 5. The shape of the observed DSC curing curve of the PETEP–CBz hybrid pre-polymer is similar to that of the DSC curing curve observed for commercial epoxy blended with benzoxazine.^42–46^ The resulting thermogram of the PETEP-biobased benzoxazine hybrid pre-polymer shows a broad exothermic peak between 179 °C and 192 °C. Further, it was observed that the bio-based cardanol benzoxazine-PETEP/polyamidoimidazoline hybrid pre-polymer shows a peak curing temperature of 185.7 °C and an enthalpy of −89.78 J g^−1^. The observed value of enthalpy in the present investigation is comparable with previously reported values of a commercial DGEBA/triethylenetetramine system (−95.6 to −98.3 J g^−1^).^47^ However, the cure temperature and enthalpy values of hybrid epoxy-benzoxazine matrices are expected to always be slightly higher than those of commercial epoxy resins because benzoxazine curing proceeds by ring opening, which requires high thermal energy. Further, hybridisation of benzoxazine with epoxy is expected to have a number of advantages in terms of enhanced properties and processing behaviour.

**Fig. 6:**
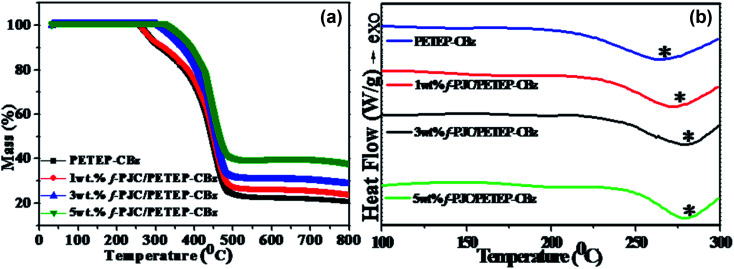
TGA (a) and DSC (b) thermograms of the PETEP–CBz matrix and the f-PJC/PETEP–CBz composites with different weight percentages.

The TGA curves of the PET waste-based epoxy/bio-phenolic benzoxazine blended matrix (PETEP–CBz) and *Prosopis juliflora* carbon-reinforced PET epoxy/bio-phenolic benzoxazine (f-PJC/PETEP–CBz) composites are shown in Fig. 6a. From the TGA results (Fig. 6a), it was found that the 5 wt% f-PJC/PETEP–CBz composite possesses a higher char yield of 38.37% than the other samples; it is expected to be more thermally stable and also function as a flame retardant composite.^48^ The temperatures at which 5% weight loss occurs for the PETEP–CBz blended matrix and 5 wt% f-PJC/PETEP–CBz composite are 278.5 °C and 354.0 °C, respectively (Table 1). The maximum stability of the PETEP–CBz blended matrix and different weight percentages of f-PJC/PETEP–CBz composites are 404.3 °C (PETEP–CBz), 442.5 °C (1 wt% f-PJC/PETEP–CBz), 447.0 °C (3 wt% f-PJC/PETEP–CBz) and 451.5 °C (5 wt% f-PJC/PETEP–CBz). The reason for the increased thermal stability and char yield may be the secondary reactions that occur during the formation of Mannich-based methylene bridges, which help to retard the decomposition.^49^ In addition, the thermal stability of the composites was improved by the reinforcing f-PJC compound; this is due to the mass barrier effect, which prevents the volatilization of polymer composites.^50^

**Table 1:** Thermal data and contact angles of the PETEP–CBz blended matrix and f-PJC/PETEP–CBz composites

S. no.	Sample	*T*_g_ (°C)	*T*_5_ wt loss% (°C)	*T*_10_ wt loss% (°C)	Char yield% at 800 °C	Contact angle (°)
1	PETEP–CBz	272.3	278.5	301.9	21.60	97.7
2	1 wt% f-PJC/PETEP–CBz	278.8	284.3	317.7	24.98	105.1
3	3 wt% f-PJC/PETEP–CBz	283.8	329.3	369.4	30.39	108.9
4	5 wt% f-PJC/PETEP–CBz	285.0	354.0	396.4	38.37	111.3

The glass transition temperature (*T*_g_) (Fig. 4b) increased with increasing weight percentage of f-PJC in the PETEP–CBz blended matrix (Table 1). The 5 wt% f-PJC/PETEP–CBz composite shows a *T*_g_ value of 285 °C, which is 13 °C higher than that of the PETEP–CBz matrix; this is due to the formation of a 3D network structure between the matrix and bio-carbon reinforcements, which in turn restricts the segmental motion of the PETEP–CBz polymer blends with increasing weight percentage of the f-PJC sample.^51,52^

### 3.5 Mechanical analysis of the PETEP–CBz blended matrix and f-PJC/PETEP–CBz composites

**Fig. 7:**
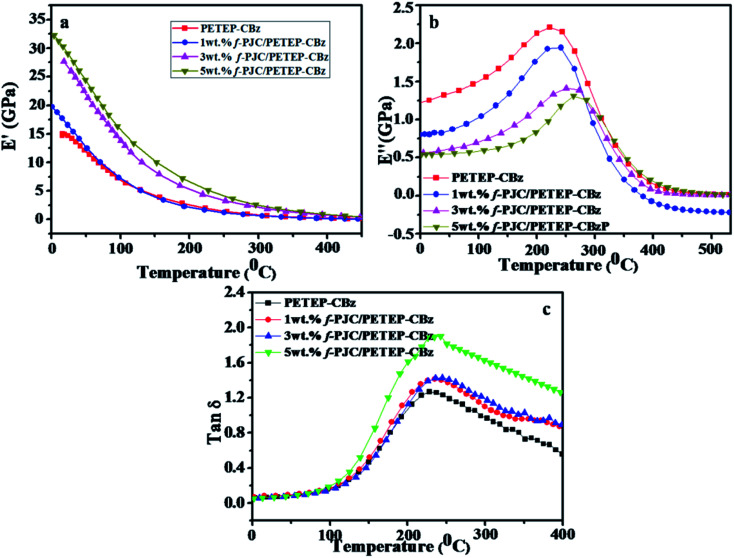
(a) Storage modulus (*E*′), (b) loss modulus (*E*′′) and (c) tan delta of the PETEP–CBz matrix and f-PJC/PETEP–CBz composites.

Fig. 7 shows the DMA curves of the storage modulus, loss modulus and tan *δ* with respect to temperature for the PETEP–CBz blended matrix and f-PJC/PETEP–CBz composites (Table 2). From the storage modulus (*E*′) graph, it is clear that the storage modulus of the PETEP–CBz matrix increased with increasing content of bio-carbon (f-PJC), which may be due to the increased cross-linking density conferred by the reinforcement. Further, the following eqn (1) was used to calculate the cross-link densities (*ν*_e_) of the PETEP–CBz blended matrix and f-PJC/PETEP–CBz composites.^53^1
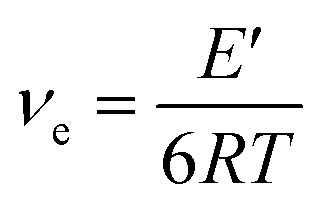
where *ν*_e_ is the cross-linking density of the PETEP–CBz matrix or f-PJC/PETEP–CBz composite, *E*′ is the storage modulus of the cured PETEP–CBz matrix or f-PJC/PETEP–CBz composite, *T* is the absolute temperature and *R* is the universal gas constant.

**Table 2:** Dynamic mechanical analysis of the PETEP–CBz matrix and f-PJC/PETEP–CBz composites

S. no.	Sample	*E*′ (GPa)	*E*′′ (GPa)	tan delta	Cross link density (J mol^−1^ K^−1^) × 10^7^
1	PETEP–CBz	1.128	2.184	1.273	2.5
2	1 wt% f-PJC/PETEP–CBz	1.646	2.165	1.422	3.3
3	3 wt% f-PJC/PETEP–CBz	2.213	1.399	1.438	4.5
4	5 wt% f-PJC/PETEP–CBz	2.591	1.299	1.683	5.1

The cross-linking densities calculated from eqn (1) for the cured PETEP–CBz matrix and f-PJC/PETEP–CBz composites are presented in Table 2. From the results (Table 2), it can be concluded that the cross-link density of the cured composites increased with increasing weight percentage of f-PJC. The loss factor is related to the degree of curing and the cross-link density.^54^ The value of the loss modulus decreases (Fig. 7b) from 2.184 to 1.299 GPa because the f-PJC/PETEP–CBz composites continue to achieve higher degrees of cross-link density with increasing weight percentage of f-PJC in the PETEP–CBz blended matrix.

The tan *δ* of a cross-linked polymer is mainly dependent on its chain segments, chemical structure and cross-link density. From Fig. 7c, it can be seen that the cured PETEP–CBz blended matrix and all the f-PJC/PETEP–CBz composites displayed tan *δ* values ranging from 1.273 to 1.683, which indicates the suitability of the composites developed in the present study for a wide range of applications. The 5 wt% f-PJC/PETEP–CBz composite exhibits the highest tan *δ* value of 1.683, reflecting its higher cross-link density. The PETEP–CBz molecular structure may be expected to contribute more flexibility through the formation of intramolecular and intermolecular forces of attraction of the molecular chains in the segments of the cured PETEP–CBz matrix and f-PJC/PETEP–CBz composites. In addition to the benzoxazine polymerisation process, the epoxy polymerization process contributes to the higher degree of cross-linking density, which appears to increase the storage modulus (*E*′) and tan *δ* values. The present system exhibits a low and broad *E*′′ (loss modulus) transition, which has the ability to absorb less energy associated with impact; this is in good agreement with previous reports.^55,56^

According to tan *δ* peak value, the 5 wt% f-PJC/PETEP–CBz composite possesses a higher *T*_g_ value due the presence of more cross-linking sites of f-PJC reinforcement, which in turn restrict the free motion of the bio-phenolic moiety with substituted long aliphatic side chains and PET epoxy moieties in the blended composites.^57^ Further, the width of the tan *δ* peak replicates the structural homogeneity of the cross-linked network; the 5 wt% f-PJC/PETEP–CBz composite shows a broad tan *δ* peak, probably due to a small amount of structural inhomogeneity, which may arise from the reinforcement effect of f-PJC on the PETEP–CBz blended matrix. Therefore, the sheet-like structure as observed from the SEM images restricts the free motion of the polymer matrix and requires higher thermal energy for the occurrence of *T*_g_.^58^ Hence, improved values of *T*_g_ were noted with respect to the addition of increased f-PJC reinforcement to the PETEP–CBz blended matrix.

### 3.6 Water contact angles and anticorrosion properties of the PETEP–CBz matrix and f-PJC/PETEP–CBz composites

**Fig. 8:**
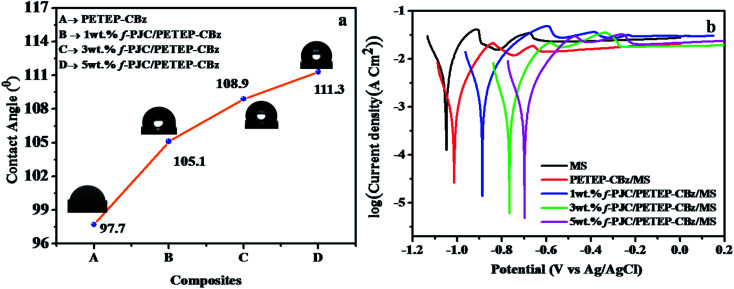
Water contact angles (a) and Tafel plots (b) of the MS plate, PETEP–CBz/MS and f-PJC/PETEP–CBz/MS electrodes.

Fig. 8a displays the water contact angles of the PETEP–CBz matrix and various weight percentages of f-PJC/PETEP–CBz composites. From Fig. 8a, it can be observed that the water droplets on the f-PJC/PETEP–CBz surface are nearly spherical in shape, suggesting its hydrophobic behaviour.^59^ The reinforcement of f-PJC also increases the hydrophobic properties of the prepared PETEP–CBz composites. From the contact angle studies, it was observed that the water contact angle increased from 97.7° to 111.3° from the PETEP–CBz blended matrix to the 5 wt% f-PJC/PETEP–CBz composite (Table 1). Further, the surface contact angles of water on the PETEP–CBz blended matrix and the f-PJC/PETEP–CBz composites with values greater than 90° indicate that the PETEP–CBz blended matrix and composites exhibit hydrophobic behaviour.^60^

The improved hydrophobic properties can also be explained on the basis of the SEM images; it was observed that the surface roughness increases upon the addition of f-PJC reinforcement to the PETEP–CBz matrix, which in turn increases the contact angle from the PETEP–CBz matrix to the 1, 3 and 5 wt% f-PJC reinforced PETEP–CBz matrices. Here, an uneven surface plays a vital role to impart superhydrophobic properties; because of the rough surface, air bubbles are caught in the peaks and valleys of the surface when a water droplet is placed on the surface. Therefore, the water droplet does not contact all the surface points. Further, due to the low free energy of the surface and the presence of air, water does not penetrate into the valleys; consequently, the surface area is decreased, which results in a decrease of friction.^39^

According to a previous report, lower surface roughness has low hydrophobic character, while high surface roughness imparts increased hydrophobic character.^61^ In this regard, enhanced roughness was noted from the SEM images after the addition of f-PJC reinforcement to the composites; this may be due to the heterogeneity of the composites with increasing addition of f-PJC content, and it can also remove air voids through a de-bonding mechanism.^62,63^ In accordance with a previous report,^64^ it was also concluded that the matrix contact angle is greater than 90°; additionally, further improvement in the roughness of the surface appears to improve the contact angle and, thus, improved hydrophobic character of the resulting composites was observed. In addition, the increased hydrophobic properties of the prepared PETEP–CBz polymer composites may be due to improved cross-linked density and decreased porosity in the polymer matrix with respect to the increased quantity of amine-functionalized *Prosopis juliflora* carbon (f-PJC) in the composite materials. Furthermore, a synergic effect resulting from the matrix-filler inter phase in the prepared composites is responsible for the improvement of different properties, including the hydrophobic properties of the prepared composite materials.

The anticorrosive performance of the coatings was evaluated by Tafel plots (Fig. 8b) obtained at room temperature (30 °C) in 3.5% NaCl solution.^65^ The system consisted of two electrode cells in which Ag/AgCl electrode served as a reference and coated/uncoated plates were used as working electrodes. The surface area of the coated/uncoated stainless steel plates immersed in 3.5% NaCl solution is 1 × 1 cm^2^ in all cases. The corrosion potential (*E*_corr_) and the corrosion current (*I*_corr_) were obtained from Fig. 8b by the extrapolation method. The *I*_corr_ of the metal substrate decreased from −2.50 μA cm^−2^ to −3.42 μA cm^−2^ from the uncoated mild steel (MS) to the 5 wt% f-PJC/PETEP–CBz composite-coated MS plate. In addition, the 5 wt% f-PJC/PETEP–CBz (*E*_corr_ = −0.69 mV)-coated MS plate showed a less negative corrosion potential (*E*_corr_) value compared to that of the PETEP–CBz coated MS plate (*E*_corr_ − 1.05 mV) and uncoated mild steel (MS) plate. The positive *E*_corr_ shift from anodic to cathodic with lower values of corrosion current can be attributed to a decrease in the oxygen concentration at the metal surface and a greater barrier effect against dissolved ions in water, which arises from the higher cross-linking density, less porous nature, compact molecular structure and better compatibility with MS plates.^66^ From the results obtained, it is suggested that the PET bottle waste-based epoxy blended bio-phenolic benzoxazine composites developed in the present work can be used as coating materials for high performance industrial applications.

## 4. Conclusion

In the present work, PET epoxy resin was synthesized from PET bottle waste and was blended with bio-phenolic benzoxazine. The PET epoxy blended bio-phenolic benzoxazine matrix was reinforced with varying weight percentages of bio-carbon (f-PJC). The PET epoxy-CBz based composites show higher values of *T*_g_ and higher storage moduli than the PETEP–CBz matrix. In addition, the bio-phenolic benzoxazine shows excellent miscibility with the PET waste-based epoxy resin. Further, a homogeneous surface with a highly cross-linked network structure was obtained for the cured PETEP–CBz matrix and f-PJC/PETEP–CBz composites. The prepared composites exhibited good thermal and mechanical properties. The PETEP–CBz matrix and its composites (f-PJC/PETEP–CBz) also showed increased *E*_corr_ and decreased *I*_corr_ values. From the results obtained from different studies, it is concluded that the hybrid composite materials developed in the present work possess excellent thermal stability, good thermo-mechanical properties, and improved surface and hydrophobic properties, including good resistance to corrosion; thus, they are useful for high performance industrial applications. In addition, the present work is considered to be an effective method of utilisation of waste materials in combination with sustainable and renewable materials for microelectronics and corrosion-resistant applications.

## Conflicts of interest

There are no conflicts to declare.

## Supplementary Material
